# 15727 K_ATP_ channel prodrugs as therapeutics for chronic pain and substance abuse disorders

**DOI:** 10.1017/cts.2021.422

**Published:** 2021-03-30

**Authors:** Alexis Doucette, Kayla Johnson, Peter I. Dosa, Amanda H Klein

**Affiliations:** University of Minnesota

## Abstract

ABSTRACT IMPACT: Pharmacological activation of K_ATP_ channels may provide analgesia and attenuate opioid tolerance and withdrawal OBJECTIVES/GOALS: Our long term goal is to develop therapeutics for the treatment of the overuse of opioids. The objective of this application is to test novel K_ATP_ channel-targeting prodrugs in rodent models of neuropathic and inflammatory pain in addition to opioid tolerance after chronic morphine administration. METHODS/STUDY POPULATION: In one study, two different measures for chronic pain were implemented in mice. Male and female mice (n=10) were subjected to spinal nerve ligation (SNL) or intraplantar injection of Complete Freund’s Adjuvant (CFA) to induce neuropathic and inflammatory pain, respectively. Administration of K_ATP_ channel prodrugs (60ug, it) attenuated mechanical hypersensitivity after SNL or CFA compared to vehicle (saline). In a separate study, changes in mechanical hypersensitivity were tested while mice undergo chronic morphine treatment (15mg/kg, 2x, 5 days) with administration of the prodrugs. Tolerance was measured as the loss of antinociception, and withdrawal is measured ˜24 hours after the final morphine injection. RESULTS/ANTICIPATED RESULTS: Intrathecal administration of either K_ATP_ channel prodrugs significantly attenuated mechanical hypersensitivity after SNL and significantly attenuated mechanical hypersensitivity after CFA in mice. We predict that intrathecal administration of these prodrugs will also attenuate morphine tolerance and withdrawal in mice. This hypothesis is based off our previous data indicating non-water soluble KATP channel agonists produce analgesia and attenuate morphine tolerance in mice. DISCUSSION/SIGNIFICANCE OF FINDINGS: Pharmaceutical strategies to utilize K_ATP_ channels for therapeutics have been hindered due to the low solubility and low ability to cross the neurovascular unit. Newly developed, water-soluble K_ATP_ channel openers could be useful pharmaceutical strategy to reduce chronic pain, opioid tolerance, and withdrawal in human populations.

